# Gender inequity in eye health: what is the impact?

**Published:** 2025-03-07

**Authors:** Lisa Johnson, Nyawira Mwangi, Heidi Chase, Preeti Dhingra, Yadira Perez

**Affiliations:** 1 Global Equity and Inclusion Lead, The Fred Hollows Foundation, Sydney, Australia; 2 Deputy Director-Academic: Kenya Medical Training College, Nairobi, Kenya; 3 Chief Program Officer: Seva Foundation, San Francisco, USA.; 4 Head Sustainability: Mission for Vision, Mumbai, India.; 5 Head of Operational Research, The Fred Hollows Foundation, Melbourne, Australia.

**Equitable access to eye care benefits all of society, not just women and girls.**

**Figure:**
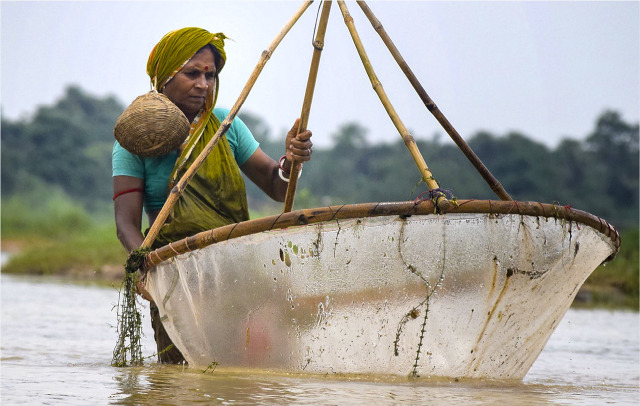
Eye health issues limit women's ability to work, which contributes to economic instability for them and their family. india

© STEVE STOCKTON, COURTESY OF IAPB CC BY-NC-SA 4.0

Improving access to eye care is essential for enhancing women's overall health and wellbeing. Vision problems can profoundly impact daily life, diminishing quality of life^1^ and increasing health risks, including those related to mental wellbeing.^2^ Women often shoulder the dual responsibilities of paid work (employment) and unpaid work (household management, caregiving responsibilities, and the associated mental load). Untreated vision issues can impair their ability to perform essential tasks, manage their health effectively, and contribute to the wellbeing of their families and communities.

When women and girls have less awareness of, and access to, eye health services, this results in delayed diagnoses, higher preventable blindness rates, increased health care costs, and reduced quality of life. Long-term effects include lower educational attainment for girls, reduced productivity, poverty, and worse health outcomes - all of which deepens existing gender inequity.

Vision impairment can have profound effects on overall health, increasing the risk of accidents and falls, and complicating the management of chronic conditions like diabetes. Untreated eye conditions can worsen over time, leading to blindness, which further restricts women's mobility, financial authority, and independence, and increases their risk of other impairments. There are also wider impacts on communities and at national level. When women and girls do not have the eye care they need, this hinders socioeconomic development, raises national health care costs, and negatively impacts community wellbeing.

“Visually impaired women are highly vulnerable to violence, crime, and assaults in private and public spaces and face challenges accessing services and support as survivors of violence.”

These effects have greater impact on women and girls as they already have worse access to health care. The financial costs to treat eye care are compounded as women and girls’ needs may not be prioritised by families, health providers, and society. There is also evidence that visually impaired women are highly vulnerable to violence, crime, and assaults in private and public spaces; they also face challenges accessing services and support as survivors of violence.^3^

## Educational and economic impact

Vision loss poses significant challenges to girls’ educational attainment, as any vision impairment can hinder their ability to read, write, and fully engage in their studies. Social stigma, discrimination, and cultural pressures often lead families to treat girls’ education as less important or urgent, especially if they have vision impairment. In some countries, social expectations of how women and girls should look are reported to discourage many girls from wearing glasses, adding to the barriers they face in achieving academic success.

Vision loss also severely limits women's ability to find work and earn an income, contributing to economic instability for themselves and their families.^4^ Vision impairment often increases women's dependence on others, adding strain to household resources. In addition, caregiving responsibilities, typically assigned to women, further restrict their economic participation. The financial burden of eye care and treatment, including travel costs, deepens this hardship, creating cycles of poverty that disproportionately impact women and their families.

## Social and emotional consequences

The psychological impact of vision impairment on women can be profound, leading to increased feelings of isolation, anxiety, and depression. Vision loss often results in decreased social interactions, as women and girls may feel embarrassed or self-conscious about their condition and their appearance. This isolation can further diminish their quality of life and reduce their participation in community activities. Additionally, the stigma associated with vision loss can deepen these challenges, making it harder to seek support.

## Broader implications for society

Women's eye health issues have broader social consequences, particularly with regards to caregiving roles and community productivity. Vision impairment reduces women's capacity to care for children or ageing parents, often shifting caregiving responsibilities to other women or even girls, disrupting their education and opportunities. These structural dynamics perpetuate cycles of poor health and economic disadvantage, as systemic discrimination leaves women with fewer resources to address vision problems or access services. When women - who are vital contributors to households and the economy - are unable to fully participate because of vision impairment, it leads to lower productivity in the community as a whole and makes inequality between generations worse.

## Conclusion

Addressing eye health disparities among women and girls is about social justice and equity, not just health care. According to global reports,^4,^^5^ investing in eye care for women not only improves individual outcomes but also yields significant economic and societal returns by enhancing productivity and reducing the economic strain on families and communities.

Prioritising gender-sensitive strategies is essential to provide women and girls with life-changing care. Stakeholders must act now to implement policies and programmes that remove barriers and foster inclusion, ensuring that no woman or girl is left behind in the fight against avoidable blindness.
